# Interplay between biofilm microenvironment and pathogenicity of *Pseudomonas aeruginosa* in cystic fibrosis lung chronic infection

**DOI:** 10.1016/j.bioflm.2022.100089

**Published:** 2022-10-22

**Authors:** Olivier Guillaume, Cosmin Butnarasu, Sonja Visentin, Erik Reimhult

**Affiliations:** a 3D Printing and Biofabrication Group, Institute of Materials Science and Technology, TU Wien (Technische Universität Wien), Getreidemarkt 9/308, 1060, Vienna, Austria; b Austrian Cluster for Tissue Regeneration, Austria; c Department of Molecular Biotechnology and Health Science, University of Turin, Turin, 10135, Italy; d Institute of Biologically Inspired Materials, Department of Nanobiotechnology, University of Natural Resources and Life Sciences Vienna, Muthgasse 11, 1190, Vienna, Austria

**Keywords:** Cystic fibrosis, *Pseudomonas aeruginosa*, Extracellular polymeric substances, Alginate, Biofilm, Antibiotic tolerance

## Abstract

*Pseudomonas aeruginosa* (PA) is a highly, if not the most, versatile microorganism capable of colonizing diverse environments. One of the niches in which PA is able to thrive is the lung of cystic fibrosis (CF) patients. Due to a genetic aberration, the lungs of CF-affected patients exhibit impaired functions, rendering them highly susceptible to bacterial colonization. Once PA attaches to the epithelial surface and transitions to a mucoid phenotype, the infection becomes chronic, and antibiotic treatments become inefficient. Due to the high number of affected people and the severity of this infection, CF-chronic infection is a well-documented disease. Still, numerous aspects of PA CF infection remain unclear. The scientific reports published over the last decades have stressed how PA can adapt to CF microenvironmental conditions and how its surrounding matrix of extracellular polymeric substances (EPS) plays a key role in its pathogenicity. In this context, it is of paramount interest to present the nature of the EPS together with the local CF-biofilm microenvironment. We review how the PA biofilm microenvironment interacts with drugs to contribute to the pathogenicity of CF-lung infection. Understanding why so many drugs are inefficient in treating CF chronic infection while effectively treating planktonic PA is essential to devising better therapeutic targets and drug formulations.

## 1 Introduction

*Pseudomonas aeruginosa* (PA) is an opportunistic and ubiquitous pathogen that occupies a wide variety of niches, from aquatic to terrestrial locations, in environments from 4 to 42 °C and in hosts ranging from plants to mammals [1]. With almost 6000 genes, the PA genome is one of the largest amongst any bacteria, which gives PA a high adaptation capability to new environmental conditions and competitive advantages over other microorganisms. One niche where PA has been able to thrive is in the lung of cystic fibrosis (CF)-patients. CF is the most prevalent genetic lethal disease in the Caucasian population, with a rate of 1 out of 3000 births [2]. Due to a reduced airway mucus clearing ability, CF-disease mainly affects lung tissues, which various microorganisms can rapidly colonize. A CF patient's lung represents an environment where most common pathogens except for PA are poorly adapted. Long-term lung colonization by PA is the predominant cause of morbidity and mortality in CF patients. Indeed, no successful antibiotic treatment exists once PA is established as microcolonies, i.e., aggregates of up to 300 μm [3], embedded within a thick mucoid biofilm matrix in the lung. The chronic inflammation and repeated infectious crises deteriorate the respiratory function of CF patients, often ending in lung transplantation. Despite medical care, the mean life expectancy of CF sufferers is reduced, ranging from 35 to 50 years [4]. The reason for the extreme antibiotic recalcitrance of PA encountered in the CF-lung is one of the most intriguing phenomena in microbiology. PA has developed intrinsic drug resistance mechanisms to many front-line antibiotics through extensive genetic adaptation [5]. It is reported that certain environmental cues, including sub-inhibitory concentrations of drugs, have been shown to transiently induce resistance, a phenomenon called adaptive resistance. The adaptive resistance mechanism has threatened the efficacy of many anti-PA treatments [6], even ones usually prescribed as “last hope” for the patients [7]. Nevertheless, the genetic trait of PA only partially explains our inability to eradicate CF-lung infection. For instance, Macleod et al. discovered that the aminoglycoside-resistance mechanisms developed by PA in CF-lung were unchanged even after six months of intensive antibiotic therapy [8]. Other researchers reported that antibiotic-resistant PA isolated from CF-biofilms regained their sensitivity to antibiotics when recultured *in vitro* [9]. Those are indications that there must be other phenotypical features governing PA's growth and drug sensitivity. Indeed, rather than possessing inherent “resistance” mechanisms, including heritable or genetic mutations, PA seems to develop additional mechanisms conferring “tolerance” to antimicrobial drugs through their CF biofilm state.

We review how the pathogenicity of PA and especially its antibiotic “tolerance” are influenced by the biofilm matrix composition of PA-infected CF lungs. Pure PA biofilms rarely or most probably never occur naturally. They live in complex ecosystems together with other microorganisms. CF biofilm infections are no exception to this rule. We are well aware that the complexity of this chronic infection cannot assume only PA as the causative agent. Other pathogen microorganisms like *Staphylococcus aureus*, *Streptococcus pneumoniae*, *Haemophilus influenzae,* or *Burkholderia cepacia* are also frequently present in the sputum of CF patients. We refer readers interested in these aspects of CF biofilms to other excellent reviews [[10], [11], [12]].

**Fig. 1:**
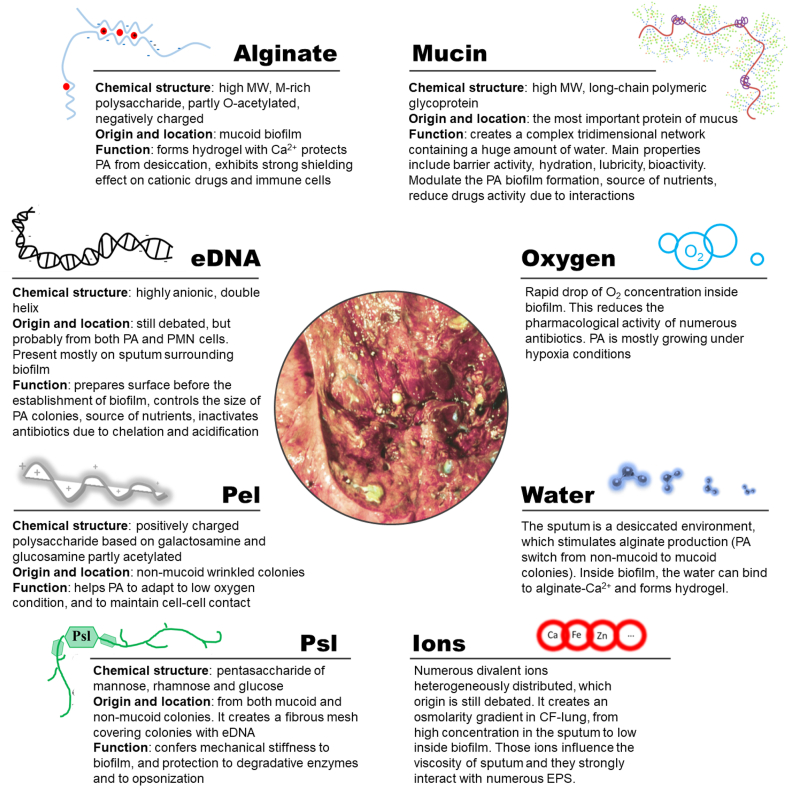
**Overview of EPS components of PA biofilm, their primary chemical features and functions.** The diversity of EPS and the specific microenvironment encountered in the lung of CF-infected patients confer high resistance to antibiotic treatment to PA. The central picture shows a lung autopsy from a PA-infected CF-lung patient, adapted with permission from Ref. [14].

Our review focuses on understanding the machinery available for PA to adapt the specific CF lung condition via compounds present in their biofilms. The biofilm represented by the extracellular polymeric substances (EPS) plays a critical role in pathogenicity, antibiotic tolerance, and long-term perseverance of PA infection [13]. EPS shields and isolates PA from physical and chemical stresses from the host and from the administered treatments. This review aims to dissect the chemical composition and role of EPS described for PA in the literature, including alginate, eDNA, Pel, Psl, and additional essential elements of the CF environment, such as mucin, ions, water, and other small molecules. Fig. 1 summarizes the chemical properties and assumed primary functions of these biofilm components.

## 2 Alginate: the centrepiece of CF mucoid biofilm

Up to 85% of the PA strains isolated from the lungs of CF patients affected with chronic infection exhibit a distinctive mucoid colony morphology [15]. This phenotype, which is generally associated with poor patient outcomes, results from initially non-mucoid PA that transition towards a specific phenotype, called “mucoid”. It is characterized by the over-secretion of one particular EPS: alginate.

**Fig. 2:**
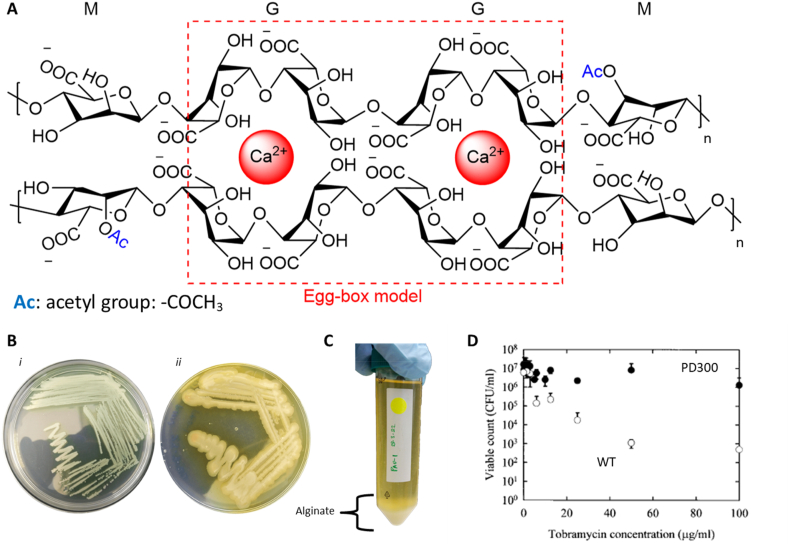
Alginate is the most abundant EPS and over-secreted by the PA mucoid phenotype. Chemical structure of alginate found in CF-biofilm and gelation mechanism with calcium ions through the “egg-box” configuration (**A**). Linker et al. estimate that (n) ranges between 700 and 2700 [16]. Photography of plates showing non-mucoid (**B**i) and mucoid PA colonies (**B**ii) grown on agar supplemented with ammonium metavanadate (AMV). Precipitated alginate chains isolated from PA mucoid colonies (**C**). The tobramycin sensitivity between non-mucoid (alginate-free PA wild-type (WT)) and mucoid PA (PD300 overproducing alginate) grown in a biofilm reactor system differ even though both microorganisms exhibit similar MIC of tobramycin of 1 μg/mL in planktonic growth (**D**) [9]. Adapted with permission from mentioned references, and photos B and C courtesy of Goodness Osondu-Chuka from the University of Natural Resources and Life Sciences Vienna.

PA-produced alginate was discovered in 1964 by Alfred Linker. He noted the presence of a polysaccharide resembling alginate in mucoid biofilms, composed of a succession of repeating β-d-mannuronic acid (M) and α-l-guluronic acid (G) units [16]. A few years later, the same author screened numerous PA variants isolated from CF-sputum and discovered that this polysaccharide has a high molecular weight (Mw from 130 up to 480 kDa) and that at least 50–90% of the repetitive motifs are M units [17] (Fig. 2A). The importance of alginate as EPS in CF-biofilm and its key role in the tenacity of the infection was nicely reviewed by May et al., in 1991 [18]. May et al. were among the first to demonstrate that the microenvironment present in CF lungs is pivotal to maintaining the mucoid phenotype, as culturing mucoid PA in vitro resulted in a spontaneous conversion to “non-mucoid” PA, producing little alginate [18]. In vitro studies have shown that alginate is not strictly necessary for PA to produce biofilms, even though non-mucoid variants exhibit a reduced capacity to adhere to surfaces. The biofilm-forming ability of non-mucoid variants, based mostly on Pel and Psl, decreases over time in the CF lung [19] and its extent is not as elevated as for mucoid variants. Secondly, CF lung infections from such variants can be medically treated, which is usually not the case once PA mutates to a mucoid phenotype.

The chemical composition of PA-produced alginate is the first intriguing feature of CF-Biofilm. Alginate is a polysaccharide of the cell walls of brown seaweeds, and it is responsible for their flexibility. Seaweeds growing in more turbulent water have higher alginate content than those living in calm zones. The chemical composition (in terms of M/G ratio) also plays a vital role in the chain rigidity, as alginate gels with low fractions of G units are more elastic than those with high fractions of G units [20]. Interestingly, alginate secreted by PA in CF-biofilm is M-enriched, and we can convincingly hypothesize that it must confer some specific advantages over G-enriched alginate. Fig. 2A shows the chemical structure of PA-alginate, which is helpful to gain insights into the benefits of M-rich over G-rich alginate for CF-biofilms.

Alginate bears a strong negative charge of two carboxylate groups COO- per monomer at pH above the pKa of the M and G units, which are 3.4 and 3.6, respectively. This high negative charge is maintained in the lung environment of CF patients, where the pH varies from 6.85 to 7.65 [21] and even further acidifies upon CF exacerbations [22]. The acidity of alginate has been proposed as one of the most important factors influencing the microenvironment of mucoid PA and its tolerance to antibiotics. As a consequence, alginate strongly binds to divalent cationic species, such as calcium (Ca^2+^), in an egg-box configuration (Fig. 2A). The result is a robust, chemically crosslinked hydrogel. Braccini et al. demonstrated that G-rich alginates bind calcium more strongly and specifically than M-rich alginates [23]. Due to the accumulation of calcium ions (amongst other metal ions) in the sputum of CF patients [24], the structure of the biofilm can be understood as an ionically crosslinked hydrogel (Fig. 2B and C). This hydrogel embeds the microcolonies of PA surrounded by a sticky and viscous mucus (called sputum), leading to gradual obstruction of the airways of the lungs [25].

The high binding capacity of alginate is not specific to divalent cations such as calcium. Many positively charged molecules, such as aminoglycoside antibiotics, also bind strongly to alginate [26,27]. Hence, the transition from non-mucoid PA to mucoid PA, with its copious alginate production, is responsible for a dramatic increase in resistance to antibiotics (Fig. 2D). Hentzer et al. illustrated this aspect and showed that PA was up to 1000-times more resistant to tobramycin when grown within a mucoid biofilm versus in a planktonic form [9]. This report was one of the first to highlight the importance of the 3D environment on PA's ability to develop mechanisms of antibiotic tolerance without becoming antibiotic-resistant via genetic mutations. Indeed, the PA recovered their normal drug sensibility when cultured back to a planktonic state.

The “shielding effect” exerted by alginate against numerous aminoglycosides results in biofilms with distinct regions; superficial parts can be saturated with antibiotics while antibiotics do not reach deeper ones. The immobilization of cationic drugs does not seem to compete with the binding site of Ca^2+^ to alginate, which involves G-units [28]. This observation means that the alginate secreted by PA can simultaneously complex divalent ions to keep the hydrogel structure and bind glycoside antibiotics to shield PA deep within the biofilm. Indeed, Heriot et al. showed that aminoglycosides preferentially bind M-rich over G-rich alginates [29]. Hence, the G units could serve the structural integrity of the biofilm via its high affinity for Ca^2+^ and other abundant divalent cations, while M units in the same alginate could serve a protective purpose without disrupting the integrity. Furthermore, it was observed that incubating gentamycin with hydrogels of Ca^2+^ - alginate resulted in the densification and dehydration of the polysaccharide chain network, leading to a stiffening of the hydrogel. The diffusion of large molecules through the alginate was greatly reduced due to the antibiotics-induced contraction of the hydrogel. Interestingly, those physicochemical changes were mainly observed for M-rich (similar to the one produced by PA) and not for G-rich alginates [29]. We published this result for gentamycin but observed the same effect for other aminoglycosides, such as tobramycin (unpublished data). This indicates a general protective physical mechanism of M-rich alginates over positively charged aminoglycosides. This could explain why CF biofilms are M-unit-rich and show exceptional resistance to the large classes of critical cationic antibiotics. The physicochemical modifications we observed when aminoglycosides bind to alginate could also confer further stress-response advantages for PA embedded in such 3D networks [29]. It is known that PA's growth and tolerance to antibiotics increase on stiff substrates [30]. The suggested mechanical feedback loop could similarly be triggered in 3D via a stiffening of the gel environment of the PA biofilm. Nevertheless, no information has been presented on how PA behaves to such stimulus once embedded in a 3D environment.

Another important characteristic described by Evans et al. is that the main difference between alginate isolated from PA and seaweed is the presence of acetyl groups bound to some of the hydroxyl groups of the alginate [17]. The O-acetylation can occur in the O-2 and O-3 positions of the M residues and substitutes 11–27% of the –OH group [31] (Fig. 2A). Other species have shown that fully acetylated EPS lose the attachment and lose their biofilm-forming capability. It is, therefore, mysterious why PA undertakes this acetylation biosynthesis. Again, it could be explained as an adaptative feature endowing CF biofilms with other specific physio-pathological benefits [31]. Adding acetyl groups to the hydroxyl groups of alginate M units creates hydrophobic pockets that could promote complementary binding mechanisms to the electrostatic binding interaction with cationic antibiotics suggested above [32]. Acetylation could potentially permit hydrophobic compounds to bind to alginate.

In addition to the possible protection against antibiotics, acetylation confers other advantages to the embedded bacteria. O-acetylation reduces recognition by immune cells and favors PA clustering, which renders them even more resistant to opsonization [33]. Another important aspect is that increasing the fraction of acetylated moieties correlates with a decreased activity of alginases [34], which might partially explain why Lamppa et al. reported that alginase's ability to disperse PA biofilms is decoupled from its catalytic activity [35]. Finally, acetylated alginate's decreased affinity to Ca^2+^ leads to decreased viscosity, increased chain flexibility, and increased ability to bind water and swell [36]. The airways of CF-patients are in a dehydrated state, which is one reason explaining CF-patients' high susceptibility to developing chronic infection. This is due mainly to the low lung clearance capability of the viscous mucus [37], the altered cilia beat, the obstruction of the airway, and, finally, the formation of an environment representing a perfect nidus for biofilm infection. As observed for other Pseudomonas strains (e.g., P. fluorescens, living in the soil), alginate protects from desiccation and osmotic stress [38]. Devault et al. demonstrated that a desiccated milieu favors biofilm formation and stimulates non-mucoid PA to switch to an alginate-producing mucoid phenotype [39]. Dehydration simulated in vitro by supplementing the medium with a small amount of ethanol (2%) was shown to increase the biofilm formation of PA mucoid strains by stimulating alginate production [39,40]. In addition, some PA strains are able to switch phenotype from mucoid to non-mucoid back and forth by supplementing or depleting ethanol in the medium, which is not observed for other bacteria like E. coli [40]. To summarize, upregulating alginate secretion seems to be a perfect answer for PA encountering a hostile milieu such as a desiccated lung, as this EPS can retain large amounts of water and simultaneously sequester multiple types of antibiotics once crosslinked with calcium. It can consequently play a key role in controlling the osmolarity, hydration, and antibiotic efficacy inside the biofilm environment.

## 3 eDNA: an on-demand releasable EPS to control the bacterial population

**Fig. 3:**
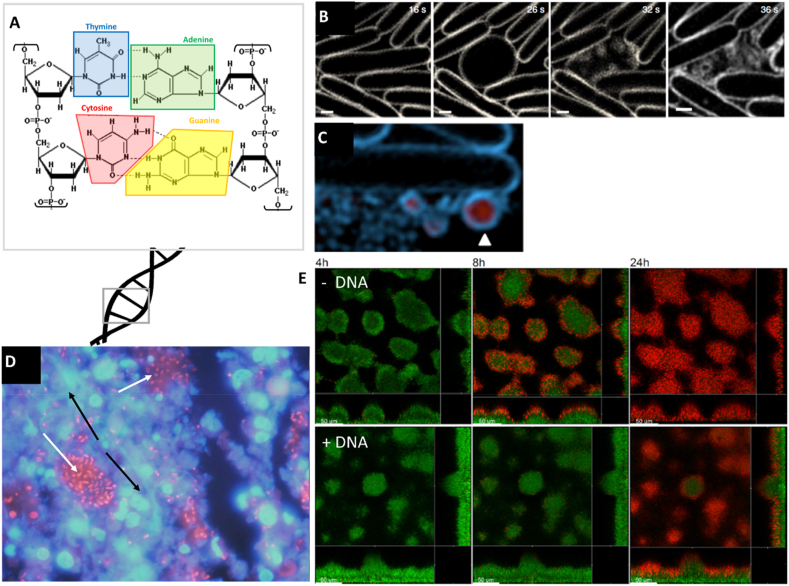
eDNA is a multi-purpose EPS. Chemical structure of double-stranded DNA (**A**). Time-lapse illustrations of the morphological changes experienced by PA upon “explosion”, from rod to cocci-like shape and release of microvesicles (shown by the white arrow with DNA appearing in red and PA cell membrane in blue) (**B** and **C**) [49]. The majority of eDNA in CF-biofilm (black arrows marking DNA stained in blue) is not colocalized with PA microcolonies (white arrows marking PA stained with FISH in red), suggesting that eDNA originates at least partially from host cells like polymorphonuclear leucocytes (**D**) [50]. Time-lapse viability assay (Live and Dead staining) of PA biofilm treated with tobramycin at 25 μg/mL, supplemented or not with exogenous DNA, showing the antibiotic tolerance effect of this EPS (**E**) [51]. Adapted with permission from mentioned references. (For interpretation of the references to color in this figure legend, the reader is referred to the Web version of this article.)

Analyzing the composition of CF sputum reveals that another EPS is copiously present in PA biofilm: the extracellular DNA. The presence of eDNA in biofilms is not a trademark for CF-chronic infection, as it is an omnipresent EPS in almost all bacterial biofilms [41]. For CF-patients affected by chronic infection, concentrations of eDNA in sputum have been estimated to be between 2 and 20 mg/mL [42,43]. eDNA represents up to 5% of the dry weight of sputum, and it is also a major (if not the major) EPS of non-mucoid PA biofilm [44]. Like alginate, DNA is a highly anionic polymer due to the phosphate in the deoxyribose backbone (Fig. 3A). DNA is known as a very potent binder of cations and cationic polyelectrolytes, which includes cationic antibiotics [45]. We refer to a review by Martin Egli, which describes that many cationic species are able to strongly interact with DNA. These interactions control DNA conformation and topology [46]. This is also true for calcium, which has been shown to induce a specific conformational change of DNA chains, in a concentration-dependent manner [47], and creates molecular bridges Ca^2+^-eDNA-Ca^2+^ [48]. Conformational changes and inter-chain complexation also occur for eDNA in biofilms and is then expected to affect the rheology of the films.

The origin of eDNA in biofilms is still a matter of debate, even though its function within biofilms does not seem to depend on its origin. eDNA can be degraded by DNAse and not nuclease, which validates its double-strand nature and excludes DNA from phages [52]. In addition, PCR analyses proved the similarity of eDNA to the bacterial genome, and eDNA seems to be released from PA voluntarily through an “explosive” mechanism, including a pronounced change in cell shape and release of cytosol and membrane material [49]. The triggering signal for PA cell explosion is not entirely known, but exogenous stresses (like the presence of antibiotics) promote this phenomenon. The cell lysis explosion releases membrane fragments, which self-anneal into microvesicles (MV, Fig. 3B and C). The MVs contain genetic materials, proteins, LPS, peptidoglycans, etc. [49]. Other small molecules, such as phenazines (i.e., pyocyanin PYO) and quorum sensing (QS) molecules, are released by PA and can further stimulate DNA release. Those compounds could be used by PA to control the size of the community or as a survival mechanism to enhance its tolerance to antibiotic treatments.

A recent in vivo murine study from Alhede et al. revealed that the eDNA originates not only from the bacteria but can even predominantly originate from the host (Fig. 3D). This corroborates a report from Lethem et al. gathered on human sputum in the 90s [53]. The analysis of human samples indicated that the immune cells (i.e., polymorphonuclear leukocytes (PMNs)) in the lungs of CF patients could release a substantial portion of the eDNA. Indeed, in CF-lungs with chronic infection, PMN cells border the PA communities without being able to engulf them. As a consequence of this frustration, PMNs undergo necrosis, which releases eDNA and other molecules (such as proteins like histone, F-actin, etc.), forming a web-like structure in a process called Neutrophil Extracellular Trap (NET) [54]. The excessive formation of NETs impacts the viscosity of the CF sputum, further exacerbating the patient's respiratory function and facilitating bacterial colonization. A review from Martínez-Alemán et al. suggests that in CF patients' lungs, PA attracts PMN by secreting bacterial surface proteins, promoting NET formation. Together with the biofilm, this creates a niche for PA to persist and establish chronic infection [54].

eDNA also plays a role in the structural development of biofilms. PA uses eDNA to prepare the surface for attachment, i.e., the formation of a microcolony's stalk. Once the biofilm is established and mature, the structural and functional role of eDNA seems to decrease in importance. This was investigated in vitro, as DNase treatments are generally effective against early-stage biofilm formation but not mature biofilm [55]. Indeed, eDNA distribution studies revealed its presence in the migration zone of mature biofilms, with long strings of DNA present at the surface but not inside the biofilm (Fig. 3D). This indicates that either only a certain portion of the PA population is releasing eDNA or that the surrounding host cells are predominantly involved in eDNA secretion [50]. Structurally, eDNA has been shown to contribute to the stability of PA biofilm matrices [56]. It colocalizes with other EPS, such as Psl [56] and Pel described below, with which it complexes directly or via cation-mediation. Fibers observable by confocal fluorescence microscopy form from these interactions. Finally, one study has shown that eDNA is also associated with a low amount of eRNA (∼4%), which contributes to the viscoelastic behaviour of the extracellular matrix [57]. RNA seems to act as a crosslinker to form a stable eRNA-eDNA gel-like structure.

eDNA plays multiple roles in chronic infections by offering protection in addition to providing structural stability. eDNA has a protective effect at low concentrations but can kill PA at high concentrations. The lethal effect of DNA is similar to EDTA [45]; it chelates cations essential for cell homeostasis. This function can be reversed in vitro by adding an excess of cations in the medium. The protective role of eDNA is multiple. Firstly, eDNA is rich in phosphorous (10% w/w). Consequently, phosphorous in eDNA is a nutrient that can circumvent the lack of other nutrients like phosphates [58], as shown for PA grown in media deprived of traditional P-nutrients. Mulcahy et al. discovered that PA isolated from CF-patients was secreting a DNase to degrade eDNA into useable C, N, and P elements [59]. They demonstrated that, in a chemically defined medium lacking nutrients, the presence of DNA could restore PA growth, but that the enzyme activity requires cations like Ca^2+^ and Mg^2+^. This significantly impacts PA colony development given the abundance and availability of eDNA and the copious amount of such ions in the sputum. Secondly, eDNA can also be used by microorganisms to repair their own DNA damages, like the ones resulting from antibiotic treatments such as Mitomycin C, through a mechanism called horizontal gene transfer (HGT) [41]. HGT is a key source of genetic diversity for microorganisms and has been shown to play an important role in the emergence of antibiotic resistances for many microorganisms [60,61], including PA [62,63].

Independently of its origin, the presence of eDNA also confers CF-biofilm with antibiotic tolerance through multiple mechanisms. For instance, Chiang et al. demonstrated that DNA-deficient PA mutants are more sensitive to aminoglycoside in a flow-chamber in vitro culture system [51]. This phenomenon can be reversed by supplementing eDNA within the media (eDNA added at 40 μg/mL, Fig. 3E). As for alginate, the antibiotic tolerance of CF-biofilm conferred by eDNA molecules present on the surface of the biofilm can be the result of its ability to bind positively charged drugs [64]. Secondly, eDNA acidifies the environment, as demonstrated by Wilton et al., who showed that PA mutants overproducing eDNA formed microcolonies of lower pH than non-mutants (down to pH 5.5) [64]. This acidification resulted in impaired pharmacological activity of numerous aminoglycosides. Indeed, DNA-treated PA colonies became more resistant to many aminoglycosides, due to the activation of a specific two-component regulatory system (i.e. PhoPQ and PmrAB TCS). This acid-induced resistance phenomenon was shown to be reversible, as re-buffering DNA-containing medium restored the aminoglycoside efficacy [64].

Finally, the chelating property of eDNA is probably the most important factor contributing to its role in antibiotic tolerance. Cations like Ca^2+^ and Mg^2+^ are essential to maintain the stability of the cell membrane, but growing PA in a cation-depleted environment also triggers the expression of modified LPS. PhoPQ-regulated LPS modification results in blocking the uptake of aminoglycosides [45]. Hence, a significant antibiotic tolerance was acquired by PA grown in media supplemented with DNA, e.g., a 640-fold increase in resistance to aminoglycoside [45].

## 4 Pel: a molecular hook initiating bacterial attachment

Another glucose-rich EPS was described by Friedman et al. for PA14 mutants that cannot secrete alginate and is deficient in Psl but still form biofilms in vitro [65]. These biofilms show a peculiar macroscopic morphology. They form pellicles and consequently colonies with wrinkled surfaces. This feature is due to an EPS named Pel after the pellicle. Pel, however, is also found in PA that produce alginate and Psl, where its localization in the biofilm is complementary to that of Psl, i.e., in the periphery and the stalk. However, Pel seems to substitute for Psl in Psl-deficient strains, where it can also perform a structural role inside the biofilm despite the major differences in chemical properties between the two EPS [66].

**Fig. 4:**
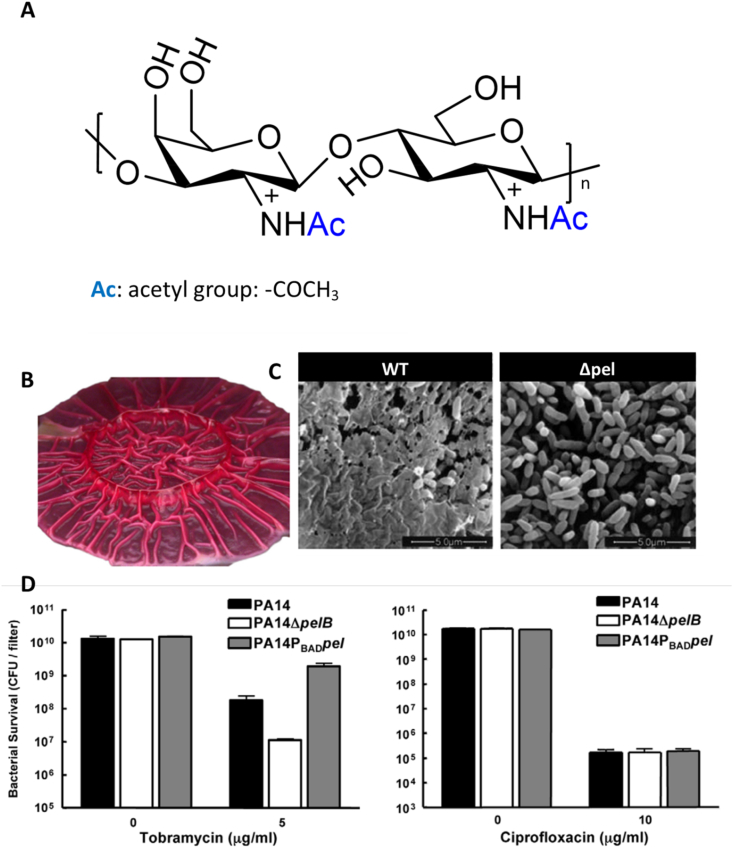
**Pel is a secreted EPS showing a wrinkling effect on PA biofilms.** Chemical structure of Pel (**A**). According to Jennings et al., n > 200 for the cell-associated Pel, and n ±2 for the secreted one [66]. Illustration of the wrinkled morphology of a Pel-rich colony grown on an agar plate (**B**) [67]. SEM pictures illustrating the microscopic differences between biofilms of a Pel-producing PA wildtype and a PA mutant lacking Pel (**C**) [65]. Demonstration of the protective effect brought by Pel against tobramycin and not against ciprofloxacin (**D**) [68]. PA14 is a mutant producing mostly Pel as EPS, able to resist tobramycin significantly better than the mutants lacking its expression (PA14ΔpelB). Stimulating the Pel production of PA14 using a mutant with an arabinose-dependent Pel secretion (PA14P_BAD_pel) further increases the tolerance to tobramycin compared to PA14. Pel affords protection only to a specific class of antibiotics, as no difference between all the mutants was observed for fluoroquinolone (i.e., ciprofloxacin). Adapted with permission from mentioned references.

Chemically, Pel is a linear and positively charged exopolysaccharide (pKa 6.7–6.9) composed of 1 → 4 glycosidic linkages of partially (around 50%) acetylated N-acetylgalactosamine (GalNAc) and N-acetylglucosamine (GlcNAc) (Fig. 4A) [3,66]. The ratio of GalNAc to GlcNAc is approximately 5:1. Two forms of Pel exist, one cell-associated with a high Mw (>80 kDa) and one secreted with an Mw of ∼500 Da corresponding to a dimer [66].

In vitro, Pel seems to play a significant role in bacterial attachment and biomass accumulation but not in further biofilm development, maturation, or maintenance [68]. The secretion of Pel is responsible for the structural heterogeneity of the biofilm's surface. The observed wrinkling was reported to be an adaptative tool to survive in the specific CF-lung environment (Fig. 4B and C). Having a wrinkled surface favors the apparition of divergent PA phenotypes. The increased surface-to-volume ratio characterizing this rough topography enhances oxygen access to cells within the bacterial community [69]. This was validated experimentally by Madsen et al., who proved that PA mutants over-producing Pel have an advantage over PA not producing Pel in a low oxygen environment [69].

The ability to improve biofilm oxygenation might be clinically relevant as CF-lungs are known as a hypoxic environment [70], due to airway lumen obstruction by viscous mucus and excessive oxygen consumption by host cells in the epithelial layer [71]. The poor oxygenation of CF-lungs is an important factor in the recalcitrance of the PA chronic infection. *In vitro* experiments revealed that oxygen barely diffuses more than 50 μm deep in PA biofilms, leading to the greater part of mature PA colonies suffering from low oxygen or even anoxia [72]. Hypoxia is responsible for most of the tobramycin tolerance [72], as the efficacy of it and many other front-line antibiotics is oxygen-dependent. PA can adapt to this lack of oxygen and even prefers building biofilm under anaerobic rather than aerobic conditions [70]. It overcomes the lack of oxygen by using NO_3_^−^ as an electron acceptor to produce ATP, explaining why copious amounts of NO_3_^−^ are present in the mucus of CF-patient.

Cell-cell interaction is also dependent on Pel secretion. In fact, daughter cells do not remain closely associated with the mother cells in aggregates of Pel-deficient PA mutants [68]. Importantly, biofilm 3D-structure hardly developed beyond a monolayer in the absence of Pel expression, indicating a crucial role in the initial PA biofilm formation, together with eDNA. The cohesion of PA biofilms also seems to depend on Pel, as it prevents disintegration *in vitro* when applying a vortex [68].

Pel does not only play a role in structurally maintaining the PA community but also in the apparition of antibiotic tolerance. Pel affords biofilms protection against certain aminoglycoside antibiotics [68], even though the mechanism requires further elucidation (Fig. 4D). The protection afforded by Pel depends on the growing phase of PA. Pel's protective role was observed only during the stationary phase, while PA in the log-phase were unaffected by the expression of Pel [73]. This could be explained by the fact that Pel secretion is mainly observed once PA is grown as a biofilm and not in the planktonic state [68]. Recently, Jennings et al. demonstrated that the positive charges of the amino groups of the deacetylated Pel moieties could strongly bind anionic species like eDNA or mucin, and the formed Pel-eDNA complex could inactivate antibiotics like tobramycin [3]. In the same work, the authors finally proved that mucolic treatment (i.e., DNAse), commonly used in clinics to improve airway clearance, was also inhibited by the Pel-eDNA complexation.

## 5 Psl: bringing mechanical protection to PA microcolonies

**Fig. 5:**
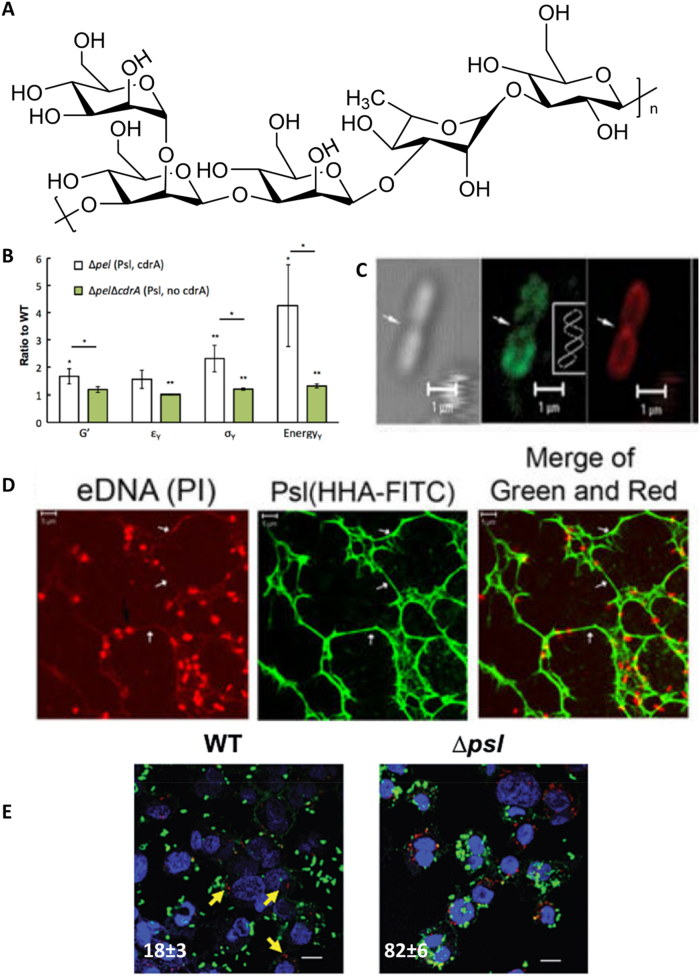
**Psl controls the mechanical behavior of PA biofilm.** Chemical structure of Psl (**A**), with n < 8 according to Byrd et al. [76]. Stiffening effect observed on Psl-secreting mutants (lacking Pel expression, Δpel), which depends on the co-expression Psl-cdrA (**B**). G′, ε_ɣ_, ơ_ɣ_ and Energy_ɣ_ indicate Young's modulus, yield strain, yield stress, and yield energy normalized against wild-type (WT) [79]. Lectin staining illustrating the helical pattern of psl surrounding PA (Psl location is visualized in green and PA membrane in red, **C**) [80]. Psl interacts with eDNA to form a mesh-like structure that maintains biofilm integrity (**D**) [56]. The presence of Psl directly impacts on the susceptibility of PA to phagocytosis, as macrophages adhere significantly more to PA lacking Psl (Δpsl) than wild-type (WT) producing Psl (**E**) [81]. The number of internalized bacteria over 100 infected cells are reported for both groups. Adapted with permission from mentioned references. (For interpretation of the references to color in this figure legend, the reader is referred to the Web version of this article.)

Another EPS which confers structural stability to the CF-Biofilm is Psl (encoded from the polysaccharide synthesis locus). Psl was first discovered in 2004 by Friedman et al. [74]. It's still debated chemical structure was first elucidated in 2007 [75]. Psl is reported to comprise a repeating pentasaccharide of 3 mannose, 1 rhamnose, and 1 glucose [76] (Fig. 5A). In contrast to the other EPS presented so far, Psl is a neutral macromolecule with branched side chains [76]. According to Byrd et al., this EPS seems to have a smaller size compared to the other EPS, between 3 and 6 KDa [76]. It is secreted by both mucoid and non-mucoid biofilm-forming PA, even though it is more present in the latter. For instance, PAO1 relies mostly on Psl to produce mature biofilm [77,78].

Jones et al. showed that treating mucoid biofilms with Psl hydrolase resulted in the total destruction of the biofilms, but only partially for non-mucoid biofilms [82]. This corroborates the observation that over-secreting alginate results in soft and weak biofilms with decreased elastic modulus, which is offset by increasing Psl synthesis. Psl-induced stiffening and increased toughness are observed only for alginate-rich biofilms [79]. The activation of the Psl machinery by PA seems to fulfill the purpose of countering the softening of the matrix when the PA transitions from a non-mucoid to a mucoid alginate-producing phenotype. Nevertheless, this effect requires the co-production of another EPS, the protein CdrA. In fact, Psl-overproducing mutants lacking CdrA have the same mechanical properties as wildtype [79] (Fig. 5B).

CdrA is a 150 KDa extracellular protein that binds to the mannose groups of Psl, and crosslinks the Psl strands. This interaction results in stiffening and provides the biofilm with mechanical stability [79]. Another cell-associated function of CdrA is to tether cells themselves, together with Psl [83]. This second interaction protects the embedded PA from mechanical and chemical damage. In fact, once CdrA binds to Psl, the complex becomes resistant to proteolytic degradation [84]. This confers a significant advantage for PA microcolonies; it protects them from the digestive effect of their own secreted proteases, which are essential virulence factors against the host organism. It is one of the numerous examples illustrating the importance of the synergistic action of multiple components secreted by PA in CF-biofilms.

The localization of Psl within a biofilm is not stable but evolves during the lifetime of the microcolonies. Ma et al. observed that Psl surrounds the PA and forms a helical pattern holding bacteria together during the biofilm initiation phase (Fig. 5C). This “cell retention” mechanism was later reported to be promoted by the lectin LecB binding to the mannose residues of Psl [85].

Upon biofilm maturation, the Psl location changes. It concentrates at the periphery of the 3D microcolonies [80], and Psl-rich zones free from bacteria are formed. The role of these zones remains unclear. They have been proposed to help bacteria from the biofilm colonize new areas or recruit new planktonic bacteria into the biofilm [86]. The term “molecular glue” is sometimes associated with Psl as it forms a fiber-like 3D mesh maintaining the biomass of the biofilm, which helps to guide the formation of microcolonies. The formation of those fiber-like structures requires the physical interaction of Psl with eDNA via hydrogen bonds [56] (Fig. 5D). This is an interesting feature as Psl has also been shown to exhibit a cytotoxic effect on human cells [82]. Being able to release Psl confers PA the ability to trigger on-demand cell lysis and consequently to control the amount of eDNA in its surroundings.

This fiber-like mesh of Psl-eDNA further protects the microcolonies against immune clearance. PA biofilms with a few hundred micrometers diameters are too big to be directly engulfed by 10 μm large neutrophils. Additionally, the Psl-enhanced stiffness of PA microcolonies is at least 10 orders of magnitude higher than the stress exerted by neutrophils during phagocytosis. This precludes that PA colonies can be broken off into small pieces by neutrophils and phagocytosed [79]. A third advantage in countering the immune system conferred by Psl to PA was demonstrated by Mishra et al. The *in vitro* and *in vivo* experiments revealed that Psl-deficient mutants were more susceptible to opsonization due to stronger complement deposition on its surface, and were consequently more easily killed by immune cells compared to wildtype of Psl++ bacteria [81] (Fig. 5E). These observations suggest that PA releasing Psl exhibit a better fitness in CF environments characterized by chronic inflammation and that Psl confers immune system protection to non-mucoid PA microcolonies. This role is taken over by alginate once the transition to mucoid colonies has occurred, a transition that can take months to years for CF patients after being colonized by PA [87].

In addition to its importance in conferring mechanical stability and immune protection to PA biofilms, Psl endows also biofilms with improved antibiotic tolerance. Billings et al. reported that Psl exhibits a key role in sequestering various antibiotics, increasing the tolerance of Psl-producing PA compared to mutants [88]. The authors demonstrated that PA overproducing Psl were more tolerant to colistin, with a 4-fold increase in its minimal bactericidal concentration for biofilms (MBC-B), compared to wild type. This protective effect was seen on young biofilms (24 h) but not on older biofilms of (48–72 h). The protective effect depended on the Psl-concentration, and it did not require the presence of the other main EPS such as Pel or alginate. Other anionic and cationic antibiotics, such as tobramycin, polymyxin, and ciprofloxacin, showed decreased efficacy against Psl-overproducing PA species. The antibiotic activity was regained once NaCl was added to the medium. Consequently, the protective mechanism was suggested to be ion-based, which excludes that Psl (being neutral) provides this protection on its own. Other charged EPS, e.g., eDNA, can interact with Psl, which could permit electrostatic interaction between drugs and the formed Psl-EPS complex [88] in a multi-stage and multi-component protective mechanism.

## 6 Mucins: a host amphiphilic network acting as a tight 3D filter

**Fig. 6:**
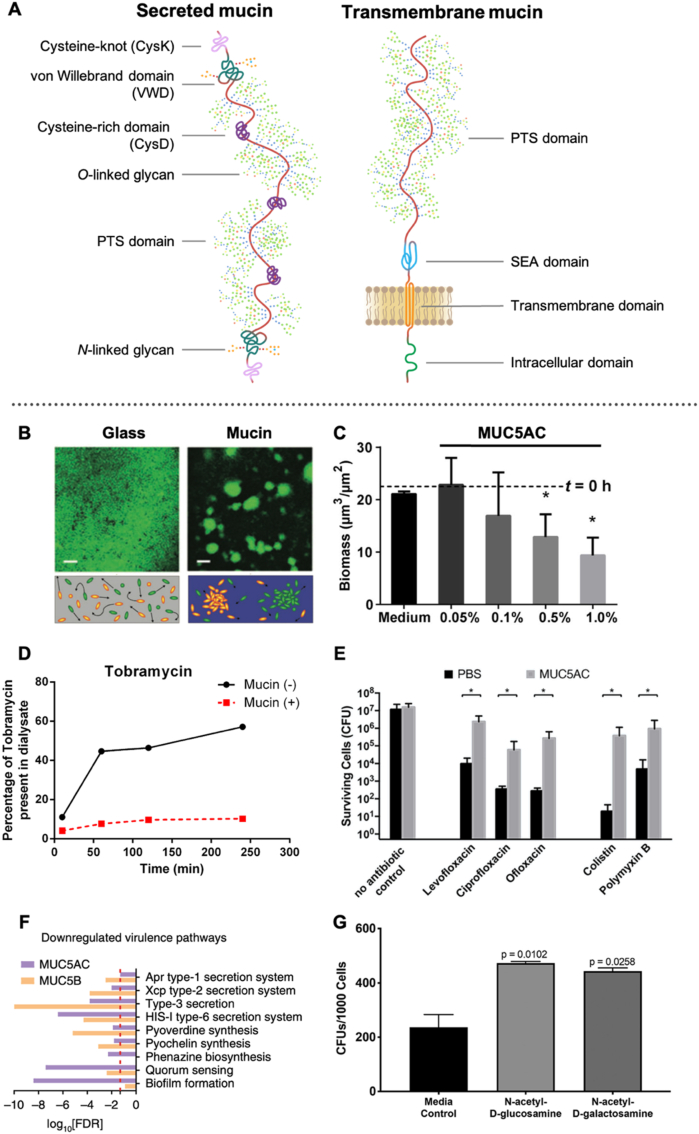
**Mucin glycoproteins build a three dimensional network around which mucus organizes.** (**A**). Schematic representation of secreted and transmembrane mucins with their most representative domains. The pink line depicts the peptide core, while the green chains represent the branched glycans. (PTS = proline, threonine, serine domain; SEA = sea urchin-enterokinase-agrin domain) (**B**) PA biofilm development on glass and mucin surfaces. PA develop a homogeneous, flat, thin biofilm on glass compared with the heterogeneous biofilm dominated by microcolonies observed on the mucin surface [97]. (**C**) Purified native mucins (MUC5AC) trigger the disruption of PA biofilms. The plot reports the biomass after 1 h of exposure to mucins. Dotted lines indicate average values of 48 h biofilms before exposure [99]. (**D**) Mucin binding reduces tobramycin antimicrobial activity. Tobramycin was measured in dialysates from samples of tobramycin that were placed into dialysis tubing with (+) or without (−) mucin [103]. (**E**) Mucin reduces the efficacy of polymyxin and fluoroquinolone antibiotics against PA. Cells were exposed to antibiotics in purified mucin (MUC5AC) for 2 h at 37 °C, and surviving cells were quantified by serial dilution and plating. Mucin samples increased the number of surviving cells [104]. (**F**) Mucin glycans expressed by gel-forming mucins (MUC5AC, MUC5B) attenuate the virulence of PA in infection by downregulating key virulence genes [117]. (**G**) The host mucin is exploited by PA to provide the monosaccharides N-acetyl-d-glucosamine and N-acetyl-d-galactosamine required for a successful infection [118]. Adapted with permissions from mentioned references. (For interpretation of the references to color in this figure legend, the reader is referred to the Web version of this article.)

The mucus, or sputum, represents a unique niche where non-mucoid PA evolves to a mucoid phenotype. The mucus organizes around mucins, a family of high molecular weight, rod-like and heavily glycosylated proteins. Mucins can be classified as transmembrane or secreted mucins, depending on whether they are anchored to epithelial cells. A rich chemical diversity characterizes both classes since they combine a long protein backbone linked to carbohydrates. Most of the amino acids within the peptide core are covalently *O*-linked to sugars called glycans, which make up to 80–90% of the protein's molecular mass (Fig. 6A) [89]. The linked carbohydrate chains are negatively charged at pH 7.4 because of the presence of sialic acid and sulfated sugars (especially galactose, *N*-acetyl-galactosamine, and *N*-acetyl-glucosamine). On the contrary, the naked aminoacidic segments rich in cysteine assemble into hydrophobic domains (*i.e.,* CysD, CysK) through covalent disulfide bonds. Mucins have been attributed a variety of properties due to their complex structure. Among them, one of the most important is their ability to form tridimensional hydrogels with high stiffness. Even though it is not yet fully deciphered how mucin hydrogels crosslink, suggested mechanisms of interactions include hydrophobic interaction [90], glycan-glycan entanglement, and calcium-mediated crosslinking [91,92]. The complex network creates a barrier against the penetration of harmful agents such as air pollutants, bacteria, and viruses. Additionally, the mucin network can hold a huge amount of water due to the mucin-associated glycans. It, therefore, provides hydration and lubrication to the underlying epithelia. Lastly, mucins act as multivalent ligands for many different cell surface receptors of endogenous cells as well as microbes [93].

The CF environment suffers from mucus stasis, airway surface liquid dehydration, and acidification. Hence it represents an ideal place for PA to grow and reproduce. Even though mucins are produced by the host and not by PA, the bacterial colonization drives the overproduction of mucus. The combination of inflammatory and immune response mediators (*i.e.*, LPS, IL-9, neutrophil elastase, TNF-α, and IL-1β) is a significant driver of mucin overproduction at the transcriptional and/or posttranscriptional level [94,95]. In physiological conditions, Amy et al*.* reported that mucin promotes rapid surface motility in PA, which has been termed “surfing” [96]. However, it appears that the CF environment modifies the mucins' conformation so that from a “rail track” mucins organizes into a solid scaffold, in which PA can initiate the biofilm formation. Indeed, studies reported that mucins could directly impact the development and function of PA biofilms, serving as an attachment surface in CF airways (Fig. 6B). For example, the PA flagellar cap protein, FliD, is responsible for mucin adhesion, and only after FliD-mediated attachment, PA starts forming biofilms with large cellular aggregates [97]. PA biofilm structures (spherical microcolonies) similar to those usually present in the CF lung have also been observed in the presence of mucin alone [3,98]. It was reported that concentrations of mucin (above 0.5%) disassemble the biofilms and disperse the cells without affecting bacterial vitality (Fig. 6C) [99]. The same effect was not observed with other viscous polymer solutions, suggesting mucins are important modulators of microbial virulence. Moreover, biofilms formed on mucin-coated surfaces have increased tolerance to the aminoglycoside tobramycin compared with biofilms grown on glass [97]. This indicates that also mucin plays a critical role in the therapeutic outcomes.

The increased tolerance of PA toward tobramycin is one of the most significant examples of how mucin and mucus alter the efficacy of drugs. Still, many drugs were reported to have their activity altered by mucus [100]. The mucin-controlled architecture of mucus might explain these findings. The three dimensional framework formed by the intricate bottle-brush structure of mucins can prevent the penetration of small compounds by a complex size- and affinity-dependent filtering activity. Mucins have a huge binding capacity because of the simultaneous presence of hydrophilic, hydrophobic, and negatively charged domains. Lipophilic drugs interact with some of the peptide parts of mucins (i.e., CysK, VWD, CysD); hydrophilic molecules engage mucins either through H-bonding or, if positively charged, by electrostatic interactions on the negatively charged carbohydrate chains [101,102]. Such a complex filtering activity can impact the efficacy of pharmaceuticals in eradicating PA infection. Huang et al. reported that polycationic drugs such as the aminoglycoside tobramycin and the polymyxin colistin can strongly bind mucin via electrostatic interactions, reducing their antimicrobial activity (Fig. 6D) [103]. Similarly, Tahoura et al*.* examined the activity of polymyxin and several fluoroquinolone antibiotics against PA in native mucus and purified mucin biopolymer environments. They found that mucus and mucin reduced the effectiveness of the investigated drugs because of the strong electrostatic and hydrophobic interactions established with mucin (Fig. 6E) [104]. The growing awareness of the importance of mucus on drug absorption has given birth to numerous models of mucus model systems. These include native collected mucus, purified mucin preparations, *in vitro* cell cultures, and intact mucosal tissues [[105], [106], [107], [108], [109], [110], [111], [112]]. More recently, *in vitro* mucus models have also been adapted to drug permeability platforms (*i.e.,* Parallel Artificial Membrane Permeability Assay - PAMPA, Phospholipid Vesicle-based Permeation Assay - PVPA) to study the effect of mucus on drug diffusion, yet maintaining a high-throughput capacity [109,113].

PA is generally unable to infect the mucus barriers of healthy individuals [114]. In healthy physiological conditions, secreted mucins undergo a massive swelling process, forming a three-dimensional network containing more than 90% water. In contrast, CF-diseased airways are characterized by mucus dehydration coupled with an overproduction of mucin. This results in airway surface liquid (ASL) with a solid content five times higher than healthy levels [115]. In CF, this hyper-concentrated mucus layer has a decreased mucin mesh size, causing an even less permeable mucus, acting as a tight filter. In physiological conditions, Ca^2+^ compacts mucin within intracellular vesicles. After secretion, Ca^2+^ cannot be correctly exchanged. Thus, the expansion of the mucins is impaired. Such changes in hydration, pH, and oxidative stress can introduce additional ionic, hydrogen, hydrophobic, and disulfide bonds. These drastic events likely induce mucin chemical and conformational alterations, which promote PA colonization. Recent observations support this hypothesis, showing that mucins encode potent signals influencing microbial gene expression and behavior. For instance, PA responds to *N*-acetyl-glucosamine, a glycan found on mucins, via a two-component response regulator [116]. Wheeler et al. determined that exposure to mucus triggers downregulation of virulence genes involved in quorum sensing, siderophore biosynthesis, and toxin secretion, rapidly disintegrating biofilms [117]. It was observed that mucin acts at various scales, suppressing distinct virulence pathways, promoting a planktonic lifestyle, reducing cytotoxicity *in vitro,* and attenuating infection in a porcine burn model (Fig. 1F). These regulatory functions were suggested to depend on glycans, which could suggest that mucin glycans are potent host-derived regulators of bacterial phenotype. In contrast, it has been shown that PA can also exploit mucins to achieve a successful infection (Fig. 6G). The mucin-derived monosaccharides *N*-acetyl-galactosamine and *N*-acetyl-glucosamine are required by PA to infect and cause damage to the host organism [118]. Even though PA is not provided with sialidase, which is needed to cleave individual monosaccharides, it might exploit sialidases released by lymphocytes or expressed by other bacteria co-hosted at mucosal level.

A deeper understanding of the structural modifications mucins undergo in pathological conditions and how the altered mucins interact with PA could provide valuable insights for developing efficient strategies to target bacterial infection.

## 7 Ions and other small molecules: solutes with multiple functions

The sputum of CF patients contains many other solutes and small soluble molecules. For instance, metal ions, including magnesium, iron, zinc, iron, calcium, and copper, were detected using ICP MS at concentrations significantly higher in CF than in non-CF patients [24]. Their origins remain unelucidated. It was hypothesized that they might be released from necrotic immune cells or local micro-vascular leakages [24]. The presence of those ions is a factor of bacterial virulence, as PA is known to respond to cations in its environment [45].

One hallmark of CF-patients' lungs is the abnormal viscosity of the airway surface liquid (ASL), which is at least partly due to the high concentration of Ca^2+^, but unaffected by other divalent cationic species like Mg^2+^ and Zn^2+^ [119]. As mentioned, this effect was ascribed to intravesicular mucin (present in the ASL), which becomes more compact upon interaction with calcium ions and increases the ASL viscosity. Additionally, Ca^2+^ triggers gelation of alginate chains via the egg-box configuration, further increasing the viscosity and elasticity of the ASL and CF biofilms.

Iron is also a powerful regulator of *P. aeruginosa* behavior, which was extensively reviewed by Smith et al. [120]. That 6% of PA transcribed genes are iron-responsive highlights its importance. Iron is essential for PA survival and influences its ability to form biofilms [120]. Nevertheless, iron is tightly regulated and restricted in the body. Pathogens like PA counteract this by secreting siderophores, small molecules able to sequester irons. Siderophore-mediated iron acquisition is an important virulent factor for many CF pathogens, with pyoverdine and pyochelin being the two most important siderophores synthesised by PA. PA is also capable of siderophore piracy, i.e., by using siderophores from other microorganisms [121]. Other ions like Zn and Cu can increase PA resistance to antibiotics like carbapenem, via a metal-inducible CzcRS two-component system regulator pathway [122].

One can postulate that the conditions encountered in infected CF-lungs are relatively heterogeneous and that the ASL environment is quite different from the one in the biofilm established deep inside the alveolar sac. Indeed, biofilms formed in CF-patients' lungs most likely encounter cation deprivation (due to interaction with anionic EPS as previously described). The ion-dependent pharmacodynamics of numerous antibiotics and the local depletion of such elements will impair their efficacy. In addition, an environment characterized by heterogeneous ion repartition will create zones where PA manifest varying antibiotic resistance [45]. In parallel, it might be reasonable to extrapolate that the resulting osmotic condition (hyperosmotic and hypoosmotic) varies in the lung depending on the anatomical location and the presence or density of a biofilm. As the osmolarity fluctuates, the PA must adapt themselves to prevent excessive dehydration or *a contrario* excessive swelling and rupture. As mentioned previously, upregulating alginate is one of the answers from PA to tackle dehydration. Another way to counter this external stress is for the bacteria to secrete highly soluble-organic solutes, called osmoprotectants [123]. For instance, osmoprotectant genes are upregulated upon osmotic pressure to maintain PA growth, resulting in an accumulation of intra-cytoplasmic protective compounds (e.g., K^+^, glutamate, N-acetylglutaminylglutamine amide (NAGGN), betaine, and hydrophilins) [124]. Another important finding from Aspedon et al. is that the rate at which PA is exposed to hyperosmolarity controls the activation of this protective mechanism. They discovered that growing PA in a steady-state osmotic stress environment (i.e., NaCl at 0.3 M) impacts the regulation of 116 genes, *versus* 431 when PA is subjected to a sudden shock of a similar final NaCl concentration [124].

Another class of small molecules, the phenazines, were only recently identified as potential key players explaining the persistence of *Pseudomonas*. Phenazines are generally found in late-stage microbial cultures and do not directly contribute to the growth of the colonies [125]. Laboratory strains of PA can secrete at least four different phenazine derivatives [126]. For instance, the phenazine pyocyanin (PYO) has been detected in the sputum of CF patients, which gives the faint blue color of this secretion. PYO is detected in 90–95% of CF-isolated PA. Its production plays an essential role in both acute and chronic PA-induced lung infections. Studies using animal models showed that PYO can activate an inflammation response on its own [127], and that PA mutants unable to secrete PYO induce an attenuated inflammation and less severe pneumonia [128]. Phenazines are strong oxidants, and they produce several peroxide species by reacting with oxygen, which might help PA to survive in this particular condition while being toxic to competing organisms. Another role of PYO was given by Das et al. [129]. Their work revealed that PYO could intercalate eDNA chains to a similar degree as some anti-cancer drugs and modify its double helix structure. Inhibiting DNA-pyocyanin interaction was also possible by using anti-oxidant agents such as glutathione or ascorbic acid, opening a potential new therapeutic venue to regain ASL fluidity.

**Fig. 7:**
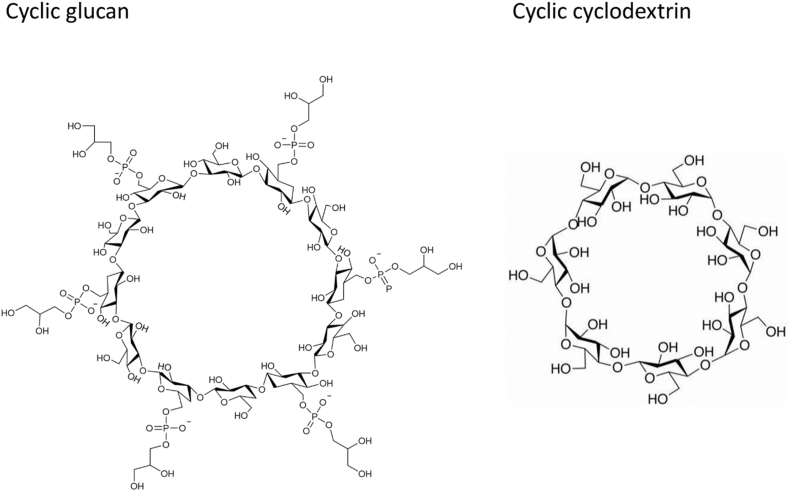
**Cyclic glucan EPS with cyclodextrin-like host-guest properties.** A comparison of the cyclic chemical structures of cyclic glucans and cyclodextrins. The central cavity could form inclusion complexes with numerous guest compounds like antibiotics, reducing their efficacy.

Polysaccharides, other than Pel, Psl or alginate have been detected in PA biofilms. With only 12 to 16 molecules of glucose arranged in a cyclic pattern, a periplasmatic glucan was first discovered on PA by Mah et al., in 2003 [130] and further characterized by Sadovskaya et al. [131]. This oligosaccharide bears an anionic charge because half of the glucose units are substituted with phosphoglycerol moieties (Fig. 7). Its chemical structure and properties are close to cyclodextrins (Fig. 7), which gives them the ability to form inclusion complexes with hydrophobic or charged guest molecules. Inclusion complexes formed with drugs, e.g., tobramycin, could slow their diffusion in the biofilm or permanently deactivate them.

Finally, PA release numerous other small soluble molecules within the biofilm, such as quorum sensing (QS) molecules. Even though they play an important role in biofilm homeostasis and antibiotic response, those compounds are not discussed in this review. They are discussed in other reviews, and we refer interested readers to the following ones [132,133].

One must not forget that CF-lung infections are often polymicrobial. Indeed, 20–50% of CF patients are co-infected with PA and *S. aureus* [134]. In the CF microenvironment, these microorganisms can both compete and cooperate. For instance, PA can secure its iron access by releasing proteases lysing *S. aureus*. Alternatively, *S. aureus* can inhibit PA biofilm formation by releasing the protein SpA, which binds to PA EPS such as Psl or type IV pili. Nonetheless, SpA was also observed to decrease the susceptibility of PA to phagocytosis. The interactions in polymicrobial communities are a vast research subject going beyond the focus of this review, and we invite interested readers to these two excellent reviews [135,136] on the topic.

## 8 Conclusion

Once PA has established mucoid-biofilms in CF-patients' lungs, the infection will remain until the patient's death. The reason for such antibiotic recalcitrance remains a fundamental question for the microbiology community. As clear from this review, the body of knowledge on the physical and chemical properties of the EPS secreted by PA point to their central role in the remarkably versatile properties of CF biofilms. The ability to produce several EPS, each with different chemical structures, charges, and affinities to other EPS affords *P. aeruginosa* formidable flexibility to shape and control its environment. It provides PA the fitness to form and maintain biofilms in the hostile environment presented by the CF lung airway. PA has developed complex mechanisms to adapt and transform CF microenvironments into a prolific niche through the toolbox afforded by its particular EPS. We summarize in this review important microenvironmental factors strongly influencing the pathogenicity and the antibiotic recalcitrance of PA in CF-lung environment.

Antibiotic resistance mechanisms often focus on the ability of bacteria to adapt their machinery genetically to expel or decompose antibiotics. However, the best defense for an organism from drugs is never to be exposed. The inherent responsiveness of the EPS matrix surrounding PA in CF biofilms provides a plethora of mechanisms to prevent classic antibiotics from reaching the cells and reduce their efficacy. This review provides an overview of how scientists have started to scratch the surface of how these physicochemical defense mechanisms act. Our improving understanding of how CF biofilms respond to the exposure to various relevant molecules and environmental changes will enable us to design better treatments, including better antibiotics and adjuvants.

## Funding

This work is funded by FWF-Stand Alone “BREATH” Project, number P33226.

## Declaration of competing interest

The authors declare the following financial interests/personal relationships which may be considered as potential competing interests: Olivier Guillaume reports financial support was provided by Austrian Science Fund.

## Data Availability

No data was used for the research described in the article.
